# PP90 Horizon Scanning For Medical Device And Non-Medical Technologies In Ukraine: An Acquired Immunodeficiency Syndrome (AIDS) Case Study

**DOI:** 10.1017/S0266462325103073

**Published:** 2025-12-29

**Authors:** Oresta Piniazhko, Tamara Mahanova, Yelyzaveta Otrubchak, Valeriia Serediuk, Iryna Musiienko, Iuliia Malyshevska, Iñaki Gutiérrez Ibarluzea, Mykhaylo Babenko, Mykhailo Lobas, Olena Filiniuk Taras Lyaskovskyi, Rabia Sucu

## Abstract

**Introduction:**

Horizon scanning has become an important global tool to respond to the exponential growth in new health technologies and rapidly evolving health needs. This case study sought to gather core information needed to prioritize new HIV/AIDS technologies that have the potential to improve health system performance and to assess the prospects of using horizon scanning in Ukraine.

**Methods:**

The EuroScan Toolkit (2014) and the PRITECTOOLS prioritization tool were employed for horizon scanning. The materials for the study included international and local databases of clinical trials, patents, academic publications; international horizon scanning and early assessment databases; and websites of public and charitable organizations, among others.

**Results:**

Three new health technologies were identified: the Harmony Portable Nucleoside Analog Analyzer based on RESTRICT technology; a machine learning model-based tool for identifying patients at risk of dropping out of HIV treatment; and a messages notification service for HIV-positive people. According to the request, the selected technologies enhance both short-term and long-term adherence to antiretroviral therapy (ART), reduce the risks of ART failure and resistance, and increase the effectiveness of behavioral interventions aimed at improving adherence. These technologies are at the marketing stage, which makes their potential integration into the Ukrainian health system feasible and necessitates informing relevant decision-makers about them.

**Conclusions:**

As a result of using the horizon scanning tool according to the EuroScan Toolkit, new technologies were identified for patients with HIV/AIDS. Information was collected regarding available clinical evidence, expected clinical and organizational changes, legal, ethical, social, and environmental consequences, and economic impact. This makes it especially relevant in the context of developing a health technology assessment guideline for medical devices in Ukraine.

